# Highly diversified core promoters in the human genome and their effects on gene expression and disease predisposition

**DOI:** 10.1186/s12864-020-07222-5

**Published:** 2020-11-30

**Authors:** Hemant Gupta, Khyati Chandratre, Siddharth Sinha, Teng Huang, Xiaobing Wu, Jian Cui, Michael Q. Zhang, San Ming Wang

**Affiliations:** 1 Cancer Centre and Institute of Translational Medicine, Faculty of Health Sciences, University of Macau, Macau, SAR China; 2 Eppley Institute for Cancer Research, University of Nebraska Medical Center, Omaha, NE 68198 USA; 3 Department of Biological Sciences, Center for Systems Biology, University of Texas at Dallas, Richardson, TX 75080 USA

**Keywords:** Core promoter, Variation, 1000 genomes, Exome, eQTL, GWAS

## Abstract

**Background:**

Core promoter controls transcription initiation. However, little is known for core promoter diversity in the human genome and its relationship with diseases. We hypothesized that as a functional important component in the genome, the core promoter in the human genome could be under evolutionary selection, as reflected by its highly diversification in order to adjust gene expression for better adaptation to the different environment.

**Results:**

Applying the “Exome-based Variant Detection in Core-promoters” method, we analyzed human core-promoter diversity by using the 2682 exome data sets of 25 worldwide human populations sequenced by the 1000 Genome Project. Collectively, we identified 31,996 variants in the core promoter region (− 100 to + 100) of 12,509 human genes (https://dbhcpd.fhs.um.edu.mo). Analyzing the rich variation data identified highly ethnic-specific patterns of core promoter variation between different ethnic populations, the genes with highly variable core promoters, the motifs affected by the variants, and their involved functional pathways. eQTL test revealed that 12% of core promoter variants can significantly alter gene expression level. Comparison with GWAS data we located 163 variants as the GWAS identified traits associated with multiple diseases, half of these variants can alter gene expression.

**Conclusion:**

Data from our study reals the highly diversified nature of core promoter in the human genome, and highlights that core promoter variation could play important roles not only in gene expression regulation but also in disease predisposition.

**Supplementary Information:**

The online version contains supplementary material available at 10.1186/s12864-020-07222-5.

## Background

Transcription initiation is the gateway for gene expression. In eukaryotic cells, RNA polymerase II-mediated transcriptional initiation is regulated by the basal transcriptional machinery of cis- and trans-elements in the core promoter region surrounding the transcriptional start site (TSS). The well-known core *cis*-elements consist of TFIIB recognition element (BRE), TATA box, Initiator element (Inr), downstream promoter element (DPE) etc. and their flanking sequences. The *trans*-elements of the preinitiation complex (PIC) consist of RNA polymerase II and six general transcription factors TFIIA, TFIIB, TFIID, TFIIE, TFIIF and TFIIH [1–10]. Variation in *cis* sequences can interfere *cis-trans* interaction and therefore modulate gene expression in physiological condition; mutation in *cis* sequences can cause abnormal gene expression contributing to pathological consequences [11–16], such as the mutation in factor IX core promoter in hemophilia B and Hemophilia B Leyden [17, 18], and the mutation in *PKLR* promoter in pyruvate kinase (PK)-deficient anemia [19]. Besides, substantial core promoters are lack of the conventional core promoter elements, such as the canonical TATA box, enriched with exceptionally high STR sequences, and islands of purine/pyrimidine [20]. Therefore, studying core promoter *cis* sequence variation can help to understanding the mechanisms of gene expression regulation and to identifying pathogenic mutations contributing to diseases.

Although the basic features of core promoter have been extensively studied in very details [1, 2], genetic diversity in core promoter at the population level remain poorly understood [21]. Extensive efforts made in past decades have collected massive gene expression data under various physiological and pathogenic conditions. However, such data mainly reflect the phenotypic change of gene expression but not the genotypic change in the regulatory machinery, including core promoter. The investigation of altered regulatory machinery, either the cis sequences or the trans factors (e.g. promoters, enhancers, insulators and their binding factors), is vital for revealing the mechanism of normal physiology and disease pathology. Lack of core promoter diversity information is attributed to multiple factors. A key factor is related to the mapping difficulties of core promoter region due to the highly conserved nature of core promoter sequences. To overcome this obstacle, we developed the “Exome-based Variant Detection in Core-promoters (EVDC)” method for core promoter sequence analysis [22]. The principle of this method is to collect core promoter sequences in exome sequences derived from the 5′ UTR probes. This method provides high sensitivity and specificity to map core promoter at genome-level.

In this study, we used our EVDC method to systematically analyse core promoter diversity by using the 2682 exome data sets from 25 human populations collected by the 1000 Genome Project [23]. Our study revealed the highly diversified nature of core promoter sequences in the human genome, tested the effects of core promoter variations in gene expression, and identified the core promoter variants as the GWAS related disease traits.

## Results

### Core promoter mapping and variant calling

The 1000 Genome Project provides rich genome sequence data to study population genetic diversity for specific functional components in the human genome. Using the “Exome-based Variant Detection in Core-promoters” method, we obtained the core promoter sequences from a total of 2640 exome data sets of 25 human ethnic populations collected by the 1000 Genome Project (Supplementary Table 1). Core promoters are classified into two classes, the canonical core promoter that contains typical motifs and major TSS site(s), and the non-canonical core promoter that is lack of typical motifs and with multiple TSS sites [24, 25]. The core promoter region was set TSS +/− 100 bps in order to cover both canonical and non-canonical core promoters within the distance by exome-derived core promoter sequences [22]. We called core promoter variants from the core promoter- mapped sequences. On the condition that a variant should be present in at least two individuals per population, we identified 31,996 distinct variants in 13,586 core promoters of 12,509 genes in the 25 populations, 90.4% were reported by the 1000 Genome data but 9.4% per population on average (4.2–17.5%) were absent in the 1000 Genome data (Table 1, Supplementary Table 2).

**Table 1:** Variants called in core promoters in each population (> = 2 individuals/population)

Population	Variants	Classification(%)		Types of variation (%)			Ts/Tv ratio
		1000 Genome	Novel	Substitution	Insertion	Deletion	
YRI	11176	10441 (92.4)	844 (7.6)	9682 (86.6)	671 (6.0)	823 (7.4)	3.67
ESN	7669	7046 (91.5)	653 (8.5)	6634 (86.5)	424 (5.5)	611 (8.0)	3.39
PEL	7623	6251 (82.5)	1331 (17.5)	6705 (88.0)	418 (5.5)	500 (6.6)	3.41
IBS	7379	6751 (91.0)	667 (9.0)	6290 (85.2)	506 (6.9)	583 (7.9)	3.61
PUR	6685	6092 (91.2)	588 (8.8)	5849 (87.5)	337 (5.0)	500 (7.5)	3.77
CEU	6477	5781 (89.8)	663 (10.2)	5574 (86.1)	417 (6.4)	486 (7.5)	3.80
GIH	6387	5874 (91.2)	559 (8.8)	5492 (86.0)	426 (6.7)	470 (7.4)	3.52
CHB	6354	5688 (88.5)	730 (11.5)	5312 (83.6)	488 (7.7)	554 (8.7)	3.65
ASW	5760	5286 (91.7)	477 (8.3)	5053 (87.7)	296 (5.1)	411 (7.1)	3.60
GWD	5567	5081 (91.4)	480 (8.6)	4807 (86.3)	328 (5.9)	432 (7.8)	3.34
MSL	5072	4834 (95.4)	231 (4.6)	4531 (89.3)	237 (4.7)	304 (6.0)	3.67
PJL	4561	3999 (87.1)	589 (12.9)	3816 (83.7)	317 (7.0)	428 (9.4)	3.50
ITU	4402	3907 (88.9)	490 (11.1)	3798 (86.3)	351 (8.0)	253 (5.7)	3.33
ACB	4308	3984 (95.0)	216 (5.0)	3806 (88.3)	212 (4.9)	290 (6.7)	3.90
STU	4282	3786 (87.8)	523 (12.2)	3607 (84.2)	304 (7.1)	371 (8.7)	3.55
LWK	4245	4047 (95.8)	178 (4.2)	3839 (90.4)	153 (3.6)	253 (6.0)	3.94
BEB	3746	3228 (89.2)	404 (10.8)	3332 (88.9)	190 (5.1)	224 (6.0)	3.78
TSI	3617	3223 (89.0)	399 (11.0)	3104 (85.8)	215 (5.9)	298 (8.2)	3.85
CLM	3016	2728 (92.0)	240 (8.0)	2659 (88.2)	160 (5.3)	197 (6.5)	3.58
CHS	2995	2637 (87.7)	369 (12.3)	2526 (84.3)	216 (7.2)	253 (8.4)	3.28
CDX	2948	2576 (86.7)	391 (13.3)	2515 (85.3)	189 (6.4)	244 (8.3)	3.25
FIN	1803	1646 (91.6)	151 (8.4)	1586 (88.0)	81 (4.5)	136 (7.5)	3.94
GBR	1315	1213 (91.1)	117 (8.9)	1121 (85.2)	77 (5.9)	117 (8.9)	3.87
JPT	953	879 (92.2)	74 (7.8)	814 (85.4)	59 (6.2)	80 (8.4)	3.81
KHV	747	650 (87.3)	95 (12.7)	643 (86.1)	45 (6.0)	59 (7.9)	3.37
Ave. total	4763	4305 (90.4)	457 (9.6)	643 (86.1)	284 (6.0)	355 (7.5)	3.60

### Differences of variant distribution between whole genome and core promoter

**Fig. 1:**
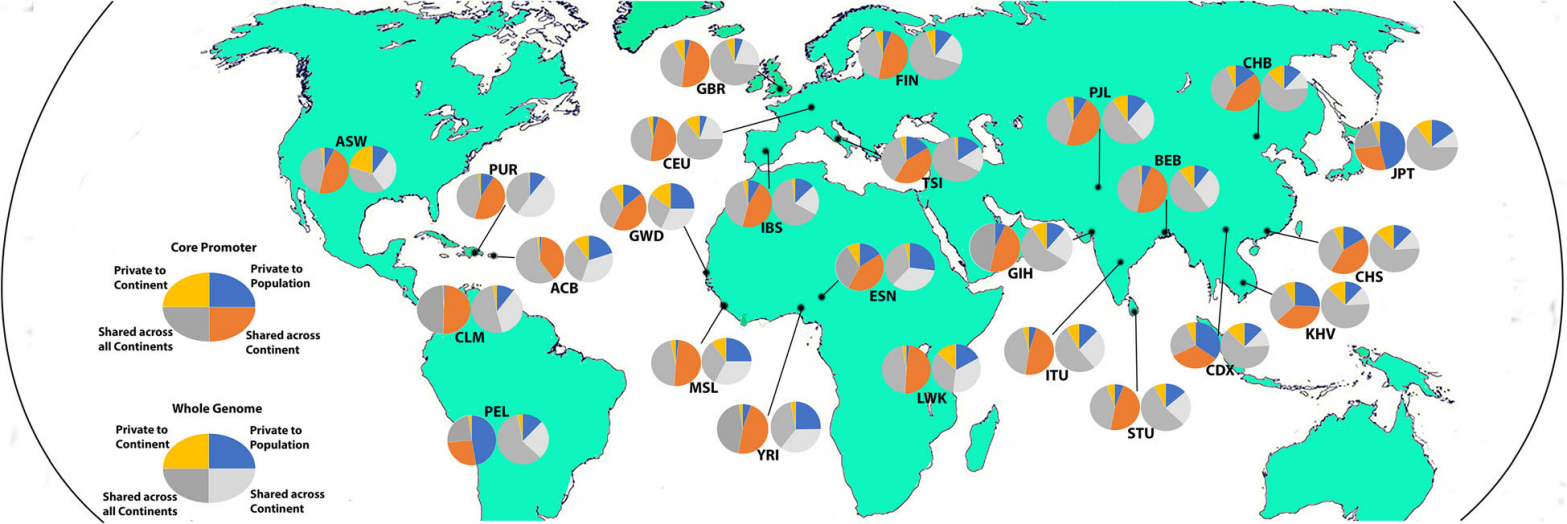
Comparison of genome variation and core promoter variation in worldwide human populations. The variants in each population are divided into four groups, including Shared across all continents, Shared across the continent, Private to continent, and Private to population. The distribution of variants is compared in pair between core promoter and whole genome. In each pair, the left circle is from core promoter variants and the right circle is from whole genome referred from Fig. 1a (1000 Genomes Project Consortium, 2015). See actual rates and *p* values in Supplementary Table 3). The original world map was downloaded from Free world maps (http://www.free-world-maps.com). The figure was generated by using Adobe Photoshop version CS6

The 1000 Genome study provided quantitative measures for the four types of genomic variants in human populations: 1) Private to population; 2) Private to continent; 3) Shared across continents; and 4) Shared across all continents. We performed a side-by-side comparison of the four types of variants between whole genome and core promoter. We observed that the proportion of each type variants were substantial differences between whole genome variation and core promoter variation in all populations except PUR (Fig. 1, Supplementary Table 3). For instance, in JPT, the ratios in the four types of variants were 15%:48, 10%:7, 10%:22, 65%:23% between whole genome and core promoter (*p* < 2.8e-^237^), accordingly. While the variants in Africa populations were well preserved in non-Africa populations reflecting human evolution history (Shared across Continent, Shared across all Continents), variants of population-specific (Private to population, Private to continent) increased in non-Africa population of East Asia and South Asia populations (Fig. 1, Supplementary Table 4). YRI had 55 variants shared in 109 of 110 individuals within the YRI population but only 6 variants as Private to YRI. In CDX, however, 19 of the 27 most common variants were Private to CDX. There were few private variants in America and Europe populations, possibly due to their closer relationship than Asia populations to the Africa ancestors.

### Differences of variation between populations

**Fig. 2:**
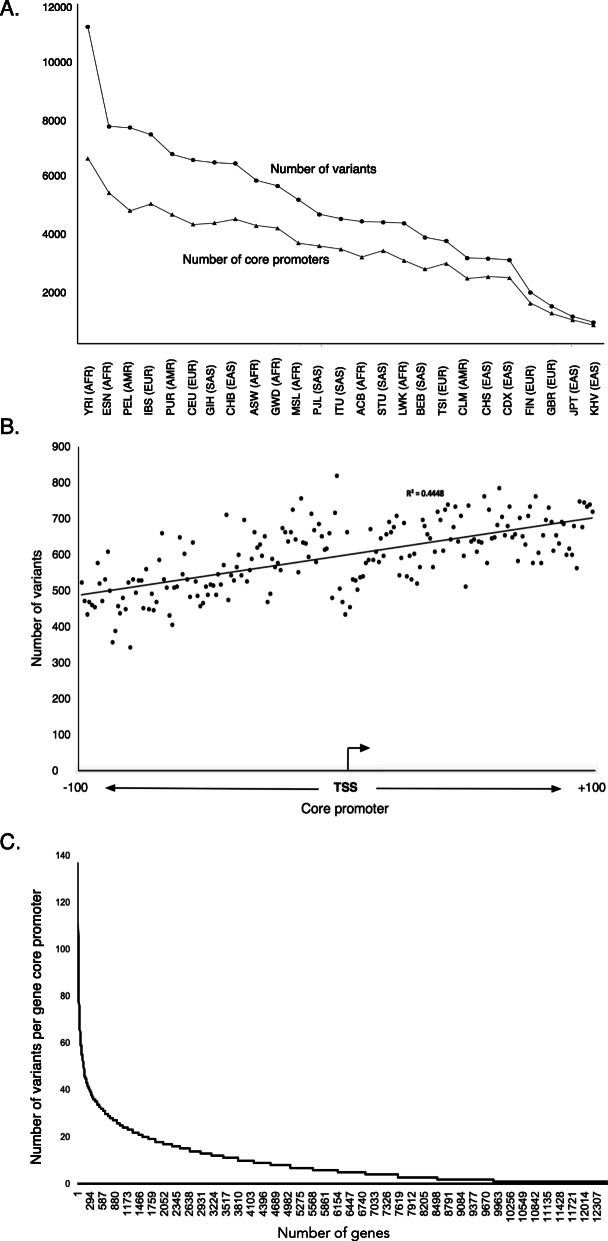
Features of core promoter variation in human populations. **a** core promoter variants and their hosting genes across human populations. It shows the highly diversified core promoter among human populations, with YRI having the highest variation frequency whereas KHV the lowest. **b**. Variant distribution across core promoter region. It shows that variation downstream of TSS site was higher than the variation upstream TSS. **c**. Variation frequencies in the core promoters of 12,509 genes containing core promoter variants

It is well determined that the African genomes are the most diversified in the humans [23]. The top 7 populations with the highest whole genome variation were ACB, ASW, ESN, GWD, LWK, MSL, and YRI (Fig. 1b in Ref. 23 1000 Genomes Project Consortium, 2015). However, data shows that this is not always the case in the core promoter region. Of the top seven populations with the highest core promoter variation (YRI, ESN, PEL, IBS, PUR, CEU and GIH), only two African populations of YRI and ESN were included (Fig. 2a). The differences imply that positive selection was stronger in core promoter than in the whole genome in reflecting the importance of altered gene expression regulation in non-Africa populations for better adaptation of their new environments after moving out of Africa. The actual numbers of variants and their affected core promoters were also substantially different between different populations as reflected by nearly 15-fold more variation in YRI than in KHV, in which YRI had 11,176 variants in 6542 core promoters whereas KHV had only 747 variants in 646 core promoters (Table 1, Fig. 2a).

### Type of variants, repetitive sequences and Ts/Tv ratio

Simple repeats are well determined to be enriched in core promoter region under evolutionary selection to regulate gene expression, and can be pathogenic as genetic predisposition for psychiatric diseases including schizophrenia [26], bipolar disorder [27]. Studies suggested that repetitive sequences can be enriched in regulatory region to play roles in gene expression regulation [25, 28, 29]. On average, 13.5% variants were insertion or deletion (Table 1). We analyzed the insertions and deletions from all the 25 populations using RepeatMasker program and determined that nearly all were simple repeats but no classical repetitive sequences of *Alu*, *B1*, and *LINE* etc. (Supplementary Table 5). Lack of these types of repetitive sequences suggest that repetitive sequences do not play significant roles in altering core promoter sequences to influence gene expression. Tv/Ts ratio have been extensively tested in exonic, intronic, intergenic, miRNA, lncRNA and whole genome but not in core promoter region [30–32]. Our calculation shows that the Ts/Tv ratio in core promoter variants were at 3.60 per population on average (3.25 to 3.90) in the 25 populations (Table 1). The rates were much higher than the 3 in the coding region and 2.0–2.1 across the genome.

### Variation upstream and downstream of TSS

The variation upstream TSS was lower than the variation downstream TSS (Fig. 2b). This feature may reflect conservation requirement in the upstream TFBSs, whereas downstream is the 5′ UTR region of coding genes, where more mutations and multiple TSSs could introduce more variation. We further compared the distribution of simple repeats. Of the 201 STRs distributed in the core promoter region, 159 were upstream but only 41 were downstream. This distribution is consistent with the previous observation [33]. Possible reason could be that long STRs in the 5’UTR region could affect on mRNA stability therefore less tolerated at the downstream.

### Functional categories for the genes with highly variable core promoters

A group of genes had high variation frequencies in their core promoters. For example, the core promoter of *PRSS1* had 26 variants, distributing 137 times in 19 populations (Fig. 2c, Supplementary Fig. 1). Interestingly PRSS1 5’UTR SNPs are known to be associated with chronic pancreatitis [34]. Using geneSCF tool, we searched 4172 genes with > = 10 core promoter variants per core promoter in the REACTOME, a database for biological pathway classification [35] (https://reactome.org). The most frequent pathway was signal transduction (492 genes). Others included metabolism, immune system, signaling by G protein–coupled receptors (GPCR), and post-translational protein modification (Table 2, Supplementary Table 6).

**Table 2:** Functional classes for genes with highly variable core promoters

Functional pathways	Number of genes
Signalling	
Cytokine Signaling in Immune system	115
G alpha (s) signalling events	225
GPCR downstream signalling	301
Olfactory Signaling Pathway	201
Signal Transduction	492
Signaling by G protein–coupled receptors (GPCR)	307
Metablism	
Metabolism	395
Metabolism of proteins	397
Metabolism of RNA	172
Immune	
Adaptive Immune System	110
Immune System	328
Innate Immune System	189
Gene expression	
Gene expression (Transcription)	231
Generic Transcription Pathway	177
RNA Polymerase II Transcription	203
Others	
Cell Cycle	116
Developmental Biology	241
Disease	150
Post-translational protein modification	232
Transport of small molecules	113
Total*	4695*

*Including genes involved in multiple functional pathways

### Variants in core promoter motifs

**Fig. 3:**
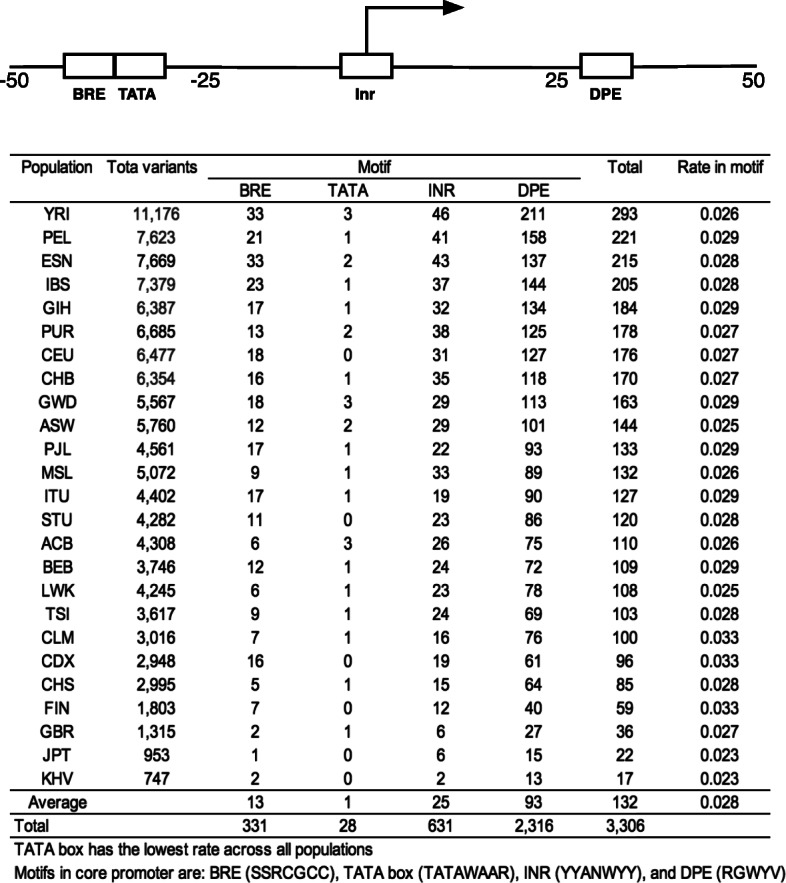
Variants distributed in the four core promoter motifs. It shows that DPE had the highest number of variants and TATA box had the lowest across all 25 populations

A total of 970 variants were located at the core promoter motifs of BRE, TATA box, Inr and DPE for 3306 times (Fig. 3). Of these four motifs, TATA box had the lowest number of variants of one per population on average, indicating that TATA box doesn’t tolerate variation as its base composition is critical for TBP (TATA-binding protein) binding to open double-strand DNA for transcription initiation. Of other three motif sequences, Inr and BRE had modest degree of variation but DPE had the highest number of variants throughout all 25 populations. This indicates that DPE is a favorable motif for variation selection to influence transcription initiation. The lower variation in TATA box, Inr and BRE is consistent with lower degree of variation upstream of TSS.

### Core-promoter variants and expression change

**Fig. 4:**
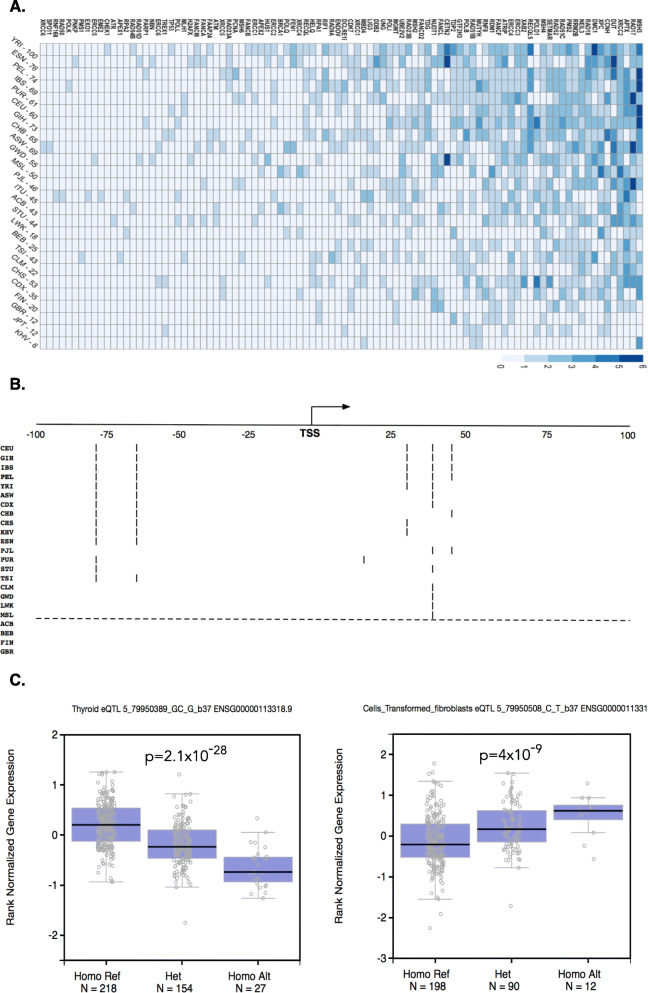
Core promoter variation in DNA damage repair genes and their effects in gene expression. **a**. Variant distribution in core promoters of DNA damage repair genes. Of the 177 genes involved with DNA damage repair, 94 genes had variants in their core promoters with the highest in YRI and the lowest in KHV. **b**. Variants in *MSH3* core promoter. Six variants were distributed 53 times in *MSH3* core promoter in 19 populations, of which 5 variants were present in 18 populations. **c**. Variants in *MSH3* core promoter alter *MSH3* expression. eQTL test showed that the variant rs151182735 at − 76 can decrease *MSH3* expression level in thyroid (*p* = 2.1 × 10^− 28^), and the variant rs1105525 at + 42 can increase *MSH3* expression level in transformed fibroblast cells (*p* = 4.0 × 10^− 9^)

The variant in core promoter is the genotype. We use Expression Quantitative Trait Loci (eQTL) to test if the genotype variation can have phenotype change as reflected by altered expression in the variant-located gene. With expression data from 53 genotyped human tissue types of 10,688 samples, the eQTL database provides a useful tool for comprehensive test of the association of core promoter variation on gene expression (https://gtexportal.org/home/tissueSummaryPage). We analyzed the entire 31,996 core promoter variants, of which 23,608 variants were identified from the 1000 Genome data, we identified 3814 core promoter variants (12%) that can cause altered gene expression (*p* < 0.05) from at least one out the 53 tissue types (Supplementary Table 7). Taking DNA damage repair genes as the example: There are 177 genes within this functional category (supplementary Table 8A). Among these genes, 94 (52%) including *MSH2*, *MSH3*, *PMS2*, *RAD51C*, *RECQL5*, *XPC* and *XRCC2* had variants in their core promoters. eQTL test showed that all the variants in the 94 genes can cause expression changes (Fig. 4a, supplementary Table 8B). For example, core promoter of *MSH3,* the gene involved in post-replicative DNA mismatch repair [36], had 6 variants, distributed in 53 individuals in 19 populations (Fig. 4b). Of the 6 variants, 5 variants could decrease *MHS3* expression whereas one (rs1105525 at + 42) might increase its expression (Fig. 4c).

### Core promoter variants as disease predispositions

**Fig. 5:**
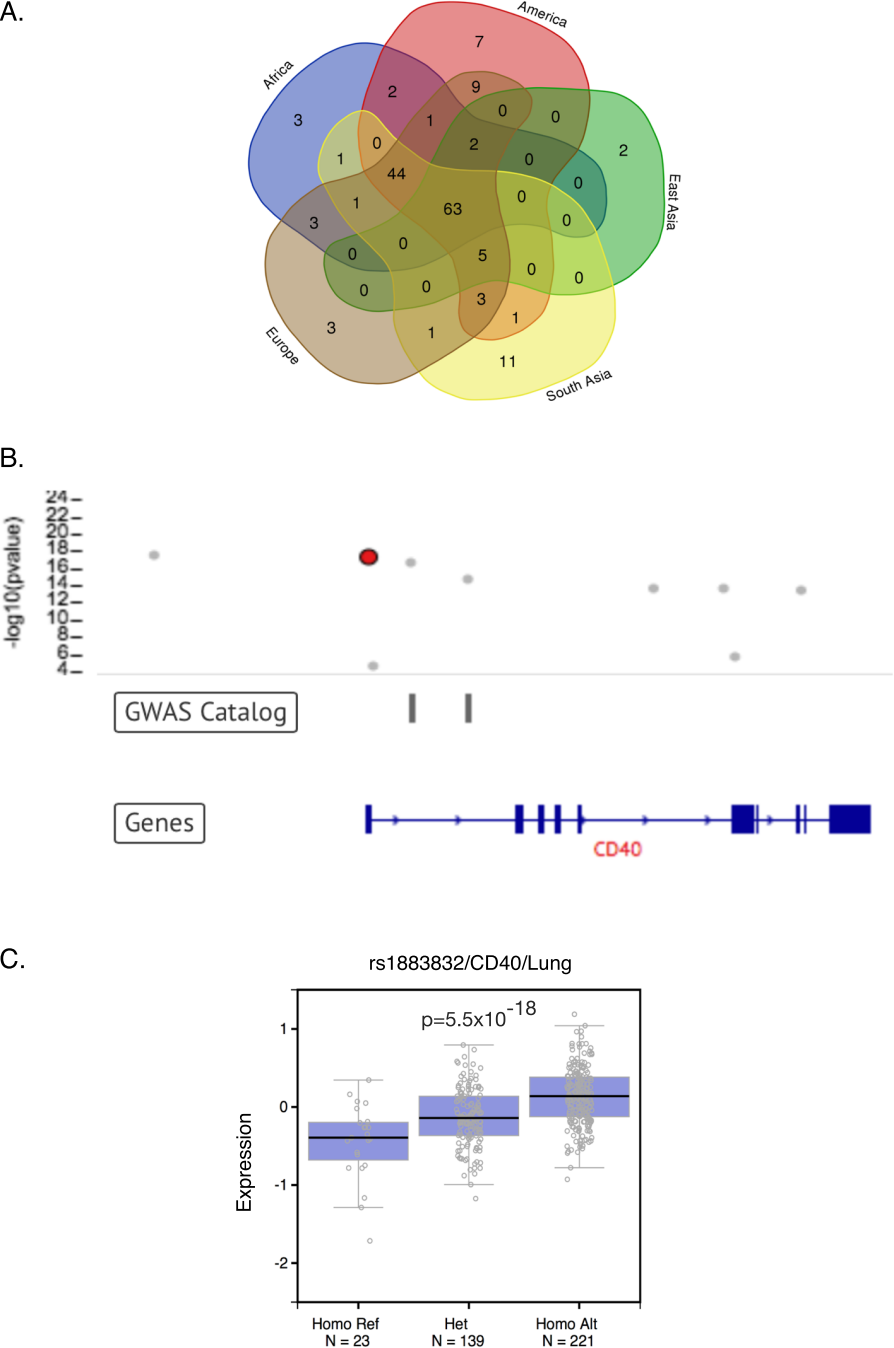
Core promoter variants associated with diseases. **a**. Venn demonstration of multiple diseases identified by core promoter variants matched with GWAS traits in different populations. Of the 163 diseases or phenotypes, 31 were shared in populations across all continents, 44 were shared in populations across the continents of Africa, American, Europe, and South Asia but not in East Asia. The rests were private to population or private to continental. **b**. Variant rs1883832 in CD40 core promoter. CD40 is a member of the TNF-receptor superfamily involving in immune and inflammatory responses. A variant rs1883832 is located at the core promoter of CD40 (+ 90). This variant was determined by GWAS study as being associated with high susceptibility to chronic hepatitis B infection in Chinese population. **c**. eQTL analysis showed rs1883832 can increase CD40 expression in lung (*p* = 5.5 × 10^− 18^)

Extensive GWAS studies have identified a large number of genetic traits associated with many human diseases, the majority are located in non-coding regions. The rich core promoter variants across diverse populations provide a resource to test if any GWAS identified disease trait loci could be located in core promoter region. GWAS catalog (the variant depository database) contains 55,297 trait loci associated with 2887 diseases or phenotypes identified from 3541 studies [37] (accessed on 8/28/2018). Searching the 31,996 core promoter variants in GWAS Catalog identified 163 (0.5%) with matched GWAS traits (0.29% of total 55,297 GWAS Catalog collection), associated with 163 diseases or phenotypes (5.6% of the 2887 GWAS analyzed diseases or phenotypes) (Supplementary Table 9A, 9B, 9C, 9D, 9E, 9F). Most of the diseases or phenotypes matched by core promoter variants/GWAS traits were present in multiple ethnic populations reflecting the common variant / common disease model of GWAS design. However, a few population-specific variants did exist, such as the variants associated with asthma present only in Africa, body mass index only in South Asia, prostate cancer (advanced) only in East Asia, dementia with Lewy bodies only in South Asia, and bone density only in Europe. Such variants suggest the presence of population-specific traits for the associated diseases or phenotypes (Fig. 5a), an issue in interpreting GWAS results [38]. Further eQTL test in the 163 variants revealed that 83 (50.9%) variants might affect the expression of the target genes (Supplementary Table 10). For examples, rs1883832 is a variant associated with susceptibility to chronic hepatitis B infection in Chinese population [39]. It is located in CD40 core promoter at − 90 (Fig. 5b). eQTL test showed that this variant could significantly increase CD40 expression in multiple tissue types, with lung as the most affected (Fig. 5c). Another example is for the germline mutations in *BRCA1* and *BRCA2*, in which germline mutation increases risk of hereditary breast cancer. As substantial cases maintain intact coding sequences, alteration of regulatory region was considered as a potential mechanism for the missing heritability [40]. The fact that only 5 variants were present in *BRCA1* core promoter in few populations and none were in *BRCA2* core promoter excludes core promoter as the mutation-targeted region.

By mining the exome data from 25 human ethnic populations, our study provides deep insight for core promoter diversity in the human genome, highlighting that core promoter variation can play more important roles than thought in gene expression regulation and diseases. An immediate use of the core promoter variation information is that it provides ethnic-based references to study core promoter variation in diseases, which can be more precise than the non-ethnic-based variation references in distinguishing between disease-causing mutation and ethnic-specific core promoter variation.

## Discussion

Although core-promoter occupies only a small part of the regulatory region, it is the gateway in controlling on-off of transcription through cis-trans interaction between core-promoter motifs and transcriptional initiation complex composed of various transcriptional factors. As the cis-trans interaction within the tight space needs to be extremely precise, a single base change in core promoter sequences could have profound impact on transcription. Our mapping analysis in 25 worldwide human ethnic populations reveals the highly diversified nature of human core-promoter sequences as reflected by the differential presence of core promoter variants between ethnic human populations. We identified the genes with highly variable core promoters, observed the effects of core promoter variation in altering gene expression, identified the functional pathways affected by the core promoter variation, and located multiple core promoter variants as the traits associated with multiple diseases identified GWAS.

The patterns of core-promoter variation distribution were substantially different from the ones of genome-wide variation distribution. As shown in Fig. 1, in all 24 populations except PUR (Puerto Ricans) population, the differences of variant distribution between genome and core promoter were highly significant in all four sections of “Private to population”, Private to continent”, “Shared across continent” and “Shared across all continents”. For example, the differences in YRI were 4.25xE^− 74^. The differential variation distribution likely reflects the consequences of evolution selection in the core promoters to fine-turn gene expression for better fitness of the ethnic population to their specific environments. Furthermore, there were substantial differences of core promoter variation between different ethnic populations as exemplified by the 15-fold more core promoter variants in YRI population than in KHV population. This may suggest that certain natural environments were much friendly for the survival of resident populations that there was not much stress to select the expression-regulatory machinery whereas other natural environments were much harsh for the resident populations that heavy selection on expression-regulatory machinery might have occurred for better adaptation of the resident populations. Among the pathways affected by core promoter variation, signal transduction pathway was affected the most, indicating the importance of signal transduction in connecting between environment stimulation and gene response for better fitness.

The relationship between core promoter variation and pathogenic consequences is particularly interesting. While core promoter variation plays important roles in adaptation, certain variation could pass the threshold of homeostasis leading to abnormal phenotypes. Most of the disease-associated traits identified by GWAS are located in intergenic region. A set of core promoter variants coincident with the disease traits identified by GWAS provides direct evidence for the involvement of core promoter mutations associated with diseases, and provides a bridge to connect GWAS-identified disease traits for functional study of their roles in disease development.

Decoding the biological basis of genetic variation in any part of the genome can use bottom-up approach of using phylogenetic, genetic diversity, segregation, and biostatistics, etc. The top-bottom approach of harvesting the “lower-handling fruits” can also be applied. In our study, we addressed the issue of core promoter diversity by using the 1000 Genome data, the first human genome data derived from representative ethnic populations across the global. Continuous efforts are needed to reach comprehensive understanding of genetic basis of core promoter variation in human population and the roles of core promoter variation in human diseases.

## Conclusions

Our comprehensive study reveals the highly diversified nature of the core promoter in human populations, and demonstrates more important roles than thoughts of core promoter variation in gene expression regulation and disease predisposition.

## Methods

### Exome data sources

The exome data generated by the 1000 Genome Project was used in the study [23] (ftp://ftp.1000genomes.ebi.ac.uk/vol1/ftp). The data contain 2640 exome data sets from 25 different ethnic populations except Mexican Ancestry from Los Angeles USA (MXL) population (Supplementary Table 1, http://www.internationalgenome.org/data-portal/sample).

### Ethic statement

The data used by the study included these from public databases including 1000 Genome Project database (ftp://ftp.1000genomes.ebi.ac.uk/vol1/ftp), GTEx database (https://gtexportal.org/home/), and GWAS database (https://www.ebi.ac.uk/gwas/). Therefore, ethical approval is not required for this study.

### Mapping core promoter sequence and calling variants

Burrows-Wheeler Aligner (BWA version 0.7.15) using mem algorithm was used to map exome sequences to the human reference genome sequences (hg19). The resulting SAM (Sequence Alignment/Map) files were converted into BAM files and sorted using Samtools utility (version 1.3.1). Duplicates were removed using MarkDuplicates and the read group information was added using AddOrReplaceReadGroups, functions of Picard tools (version 1.119), respectively. The BAM files were further processed using Genome Analysis Toolkit (GATK version 3.7) with its recommended best practices pipeline for variant calling and GATK BaseRecalibrator was used for base quality score recalibration. GATK HaplotypeCaller was used for variant calling (includes InDel realignment) in gVCF mode, followed by joint genotyping with GenotypeGVCFs, variant recalibration with VariantRecalibrator and filtration of low-quality genotypes with VariantFiltration. The called variants were further annotated using ANNOVAR for gene-based (RefSeq genes), region-based (cytoband, genomicSuperDups) and filter-based annotation (1000G, dbSNP147, ExAC, cosmic70 and ClinVar). Minimum depth for calling variants was set to 10. Core promoter sequences from the reference genome hg19 were extracted and used as the reference to identify the core promoter region as defined TSS (transcription start site) +/− 100 bps. The following consensus sequences were used for motif analysis: BRE (SSRCGCC), TATA box (TATAWAAR), INR (YYANWYY), and DPE (RGWYV). S: C/G; R:A/G; W:A/C; Y:C/T; N:A/C/G/T; V:A/C/G (IUPAC notation, International Union of Pure and Applied Chemistry) The called variants were compared to 1000 Genome, dbSNP147, and ExAC databases to distinguish between the known variants and novel variants. Novel variants were deposited in dbSNP (Supplementary Table 11). An open access database, dbHuman Core-Promoter Variation, hosting human core-promoter variation was developed for public exploration of core promoter variation data (https://dbhcpd.fhs.um.edu.mo).

### eQTL analysis and GWAS-disease associated trait analysis

For eQTL analysis, core promoter variants were searched in Genotype-Tissue Expression database [41, 42] (GTEx, https://gtexportal.org/home/) to locate their position and to determine their potential influence on gene expression. Of the mapped variants, those with the *p*-value < 0.05 were used as the representative for causing expression changes in the affected tissue type. For GWAS-disease trait analysis, core promoter variants were searched in the GWAS Catalog (https://www.ebi.ac.uk/gwas/). The identified variants were tested in GTEx for their potential effects on gene expression in human tissues. The functional categories of variant-affected genes were classified by searching the REACTOME database using geneSCF tool [43].

### Statistics analysis

Chi-squared test was used to test the distribution differences between whole genome variation and core promoter variation. The analysis was performed using SPSS 19.0.

### Availability of data and materials

All data analyzed in this study are included in this published article, its supplementary information files and database.

## Supplementary information

Additional file 1: **S1**. Exome data used in the study

Additional file 2: **S2**. Variants in each population

Additional file 3: **S3**. Variant distribution between whole genome and core promoter

Additional file 4: **S4**. Population-common and population-specific variants

Additional file 5: **S5**. Simple repeats identified in core promoter region

Additional file 6: **S6**. Functional classification of genes with high-frequent core promoter variation

Additional file 7: **S7**. Effects of core promoter variants on gene expression by eQTL analysis

Additional file 8: **S8**. Core promoter variation in DNA damage repair genes

Additional file 9: **S9**. Disease-associated core promoter variants

Additional file 10: **S10**. Effects of core promoter-located GWAS-traits on gene expression by eQTL analysis

Additional file 11: **S11**. Novel variants identified from the study

Additional file 12: **S** Figure 1. PRSS1 core promoter variation

## Data Availability

The datasets supporting the conclusions of this article are available in the URL https://dbhcpd.fhs.um.edu.mo and Supplementary Tables 1, 2, 3, 4, 5, 6, 7, 8, 9, 10, 11 and Supplementary Fig. 1. The novel variants identified from the study have been deposited in NCBI dbSNP database (Supplementary Table 11).

## Abbreviations

eQTL: Expression quantitative trait Loci
GWAS: Genome wide association study
UTR: Untranslated region
EVDC: Exome-based Variant Detection in Core-promoters
TSS: Transcriptional start site
GTEx: Genotype-tissue expression database
