# Supplementary figures and images for: Highly diversified core promoters in the human genome and their effects on gene expression and disease predisposition
Source: BMC Genomics. 2020 Nov 30;21:842. doi: 10.1186/s12864-020-07222-5 (PMC7706239; doi:10.1186/s12864-020-07222-5)

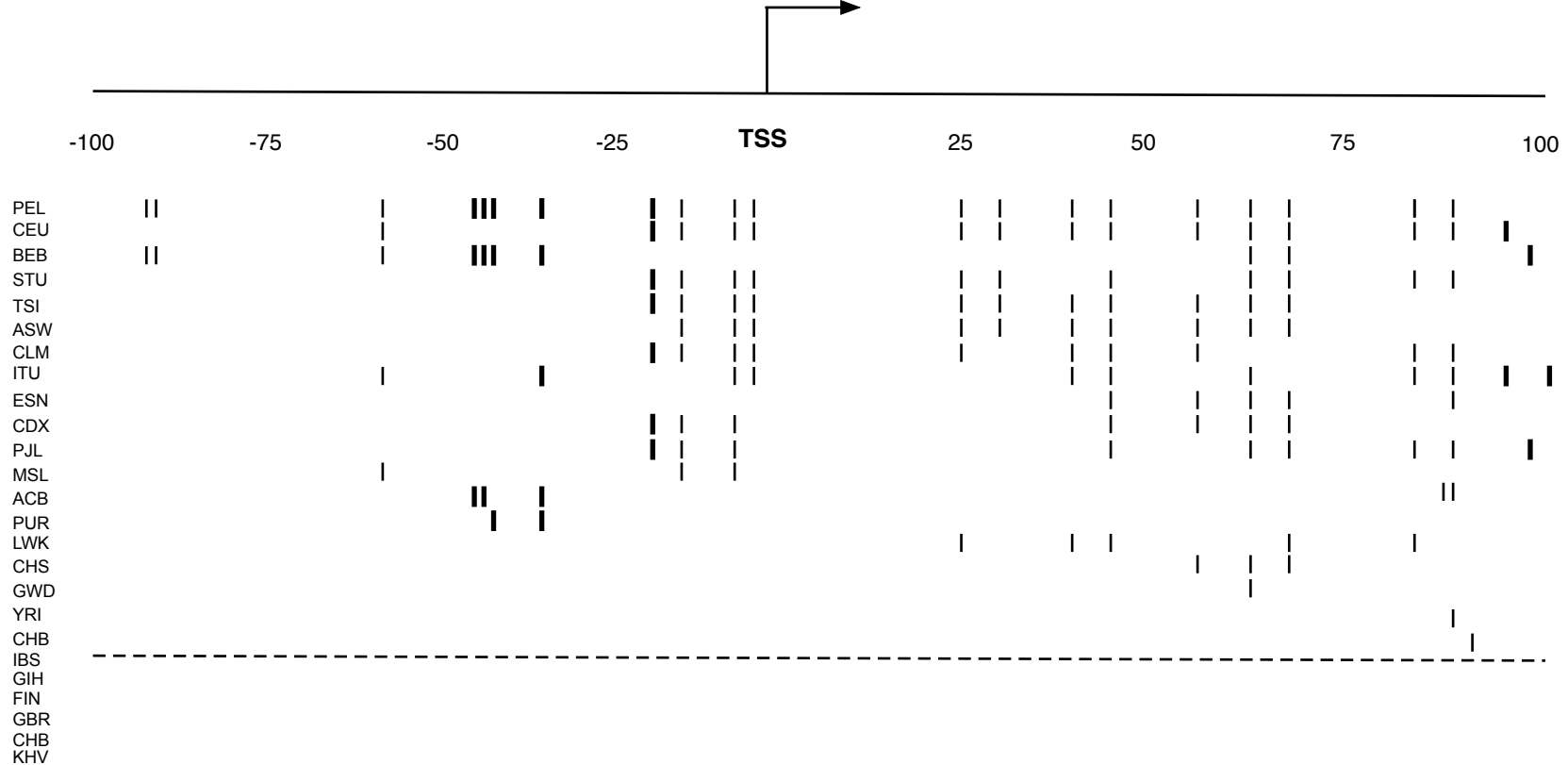

Supplement: Supplementary file 12 — S Figure 1. PRSS1 core promoter variation [file 12864_2020_7222_MOESM12_ESM.pdf]
